# CCDC74A/B are K-fiber crosslinkers required for chromosomal alignment

**DOI:** 10.1186/s12915-019-0694-9

**Published:** 2019-09-14

**Authors:** Haining Zhou, Tao Zheng, Tianning Wang, Qi Li, Fulin Wang, Xin Liang, Jianguo Chen, Junlin Teng

**Affiliations:** 1 Key Laboratory of Cell Proliferation and Differentiation of the Ministry of Education and State Key Laboratory of Membrane Biology, College of Life Sciences, Peking University, Beijing, 100871 China; 2 Peking-Tsinghua Center for Life Sciences, Academy for Advanced Interdisciplinary Studies, Peking University, Beijing, 100871 China; 3 Peking-Tsinghua Center for Life Sciences and Max-Planck Partner Group, School of Life Sciences, Tsinghua University, Beijing, 100084 China; 4 Center for Quantitative Biology, Peking University, Beijing, 100871 China

**Keywords:** CCDC74A, CCDC74B, Kinetochore fiber, Microtubule dynamic, Spindle assembly, Chromosomal alignment

## Abstract

**Background:**

Spindle microtubule organization, regulated by microtubule-associated proteins, is critical for cell division. Proper organization of kinetochore fiber (K-fiber), connecting spindle poles and kinetochores, is a prerequisite for precise chromosomal alignment and faithful genetic material transmission. However, the mechanisms of K-fiber organization and dynamic maintenance are still not fully understood.

**Results:**

We reveal that two previously uncharacterized coiled-coil domain proteins CCDC74A and CCDC74B (CCDC74A/B) are spindle-localized proteins in mammalian cells. They bind directly to microtubules through two separate domains and bundle microtubules both in vivo and in vitro. These functions are required for K-fiber organization, bipolar spindle formation, and chromosomal alignment. Moreover, CCDC74A/B form homodimers in vivo, and their self-association activity is necessary for microtubule bundling and K-fiber formation.

**Conclusions:**

We characterize CCDC74A and CCDC74B as microtubule-associated proteins that localize to spindles and are important K-fiber crosslinkers required for bipolar spindle formation and chromosome alignment.

**Electronic supplementary material:**

The online version of this article (10.1186/s12915-019-0694-9) contains supplementary material, which is available to authorized users.

## Background

The mitotic spindle, a microtubule-based machinery, is responsible for the proper chromosomal alignment and precise chromosomal segregation during cell division [1]. Kinetochore fibers (K-fibers), interpolar microtubules, and astral microtubules are the major components of mitotic spindle. Of them, K-fibers, parallel microtubule bundles, link chromosome to spindle pole and is crucial for chromosomal alignment and segregation [2]. K-fiber formation defects caused by abnormal microtubule nucleation, polymerization, or organization result in genomic instability, such as aneuploidy, which can further lead to tumorigenesis [1, 3, 4].

K-fibers are well organized and highly dynamic [1]. A large number of regulators especially microtubule-associated proteins (MAPs) are implicated in the formation and stabilization of K-fiber structure [5]. TPX2 as a microtubule-binding protein recruits protein kinase Aurora A and the motor protein XKLP2 to spindle microtubules to promote spindle pole microtubule nucleation and organization, and TPX2 also contributes to microtubule organization by directly bundling microtubules [6, 7]. HURP, a spindle assembly regulator, accumulates at spindle chromatin-proximal regions and regulates kinetochore microtubule dynamic [8–10]. NuMA, an important microtubule-crosslinking protein, abundant in spindle poles, integrates spindle pole by crosslinking microtubules [11]. NuMA forms oligomers by its C-terminus, and the oligomer captures multiple spindle pole microtubules [12]. Moreover, PRC1 only crosslinks antiparallel overlaps of microtubule plus ends in the spindle midzone at anaphase [13–15]. In addition, a few chromatin-binding factors (e.g., CHD4, ISWI, and NuSAP) have been shown to be microtubule-binding proteins and involved in K-fiber microtubule organization during mitosis [16–19]. However, none of these MAPs seems to specifically crosslink and integrate microtubules of the K-fibers.

CCDC74A and CCDC74B (CCDC74A/B), containing coiled-coil domains, are functionally unknown proteins. In this study, we reveal that CCDC74A/B are microtubule-binding proteins localized at spindles and responsible for K-fiber crosslinking and stabilization. They bind directly to microtubules through two separated microtubule-binding regions. Meanwhile, they possess self-association activity, which enables them to bundle and stabilize microtubules in K-fibers. Therefore, CCDC74A and CCDC74B are newly identified spindle microtubule regulators that promote the K-fibers stability and maintain the spindle integrity, thus ensuring proper chromosome alignment and cell division.

## Results

### CCDC74A/B are spindle-localized proteins and required for chromosomal alignment

**Fig. 1:**
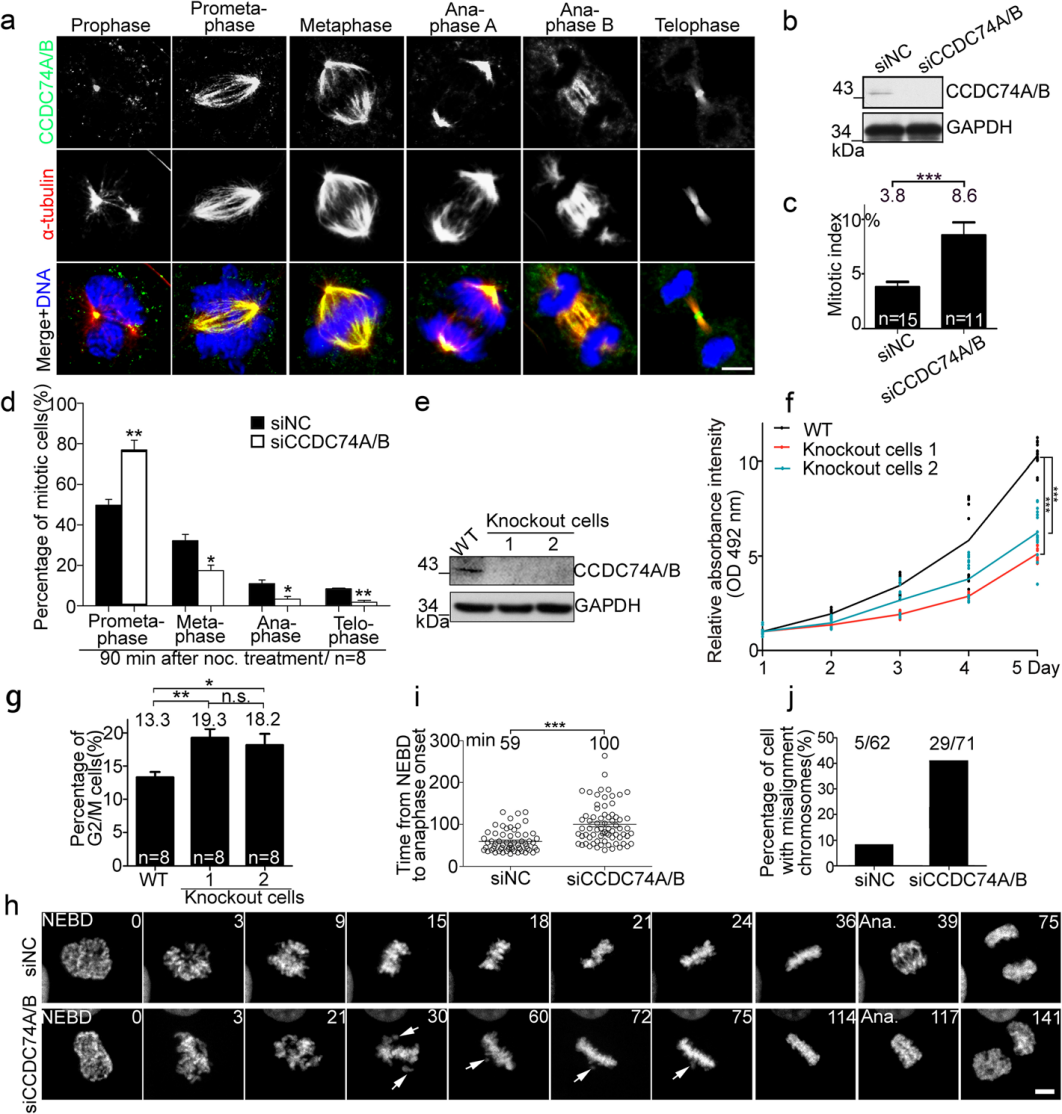
CCDC74A/B are localized at mitotic spindles and required for chromosomal alignment. **a** Immunofluorescence of α-tubulin (red) and CCDC74A/B (green) in COS7 cells. DNA was stained with DAPI (blue). Scale bar, 5 μm. **b** Western blots of CCDC74A/B in HeLa cells transfected with negative control-siRNA (siNC) or with CCDC74A/B-siRNA (siCCDC74A/B) for 60 h. GAPDH was the loading control. **c** The mitotic index of HeLa cells after siNC- or CCDC74A/B-siRNA transfection for 60 h (six independent experiments). **d** Percentages of HeLa cells in mitosis after siNC- or CCDC74A/B-siRNA transfection for 60 h, followed by 1 h nocodazole treatment (noc., 1 μg/ml) then released (6 independent experiments). **e** Western blots of CCDC74A/B in wild-type (WT) and 2 CCDC74A/B knockout HEK293T cells. GAPDH was the loading control. **f** Wild-type and 2 CCDC74A/B knockout HEK 293T cells were cultured in 96-well plates. MTT assay was performed at daily intervals over 5 days (6 independent experiments). **g** Flow cytometric analysis of the percentages of wild-type and 2 CCDC74A/B knockout HEK293T cells in G2/M phase (6 independent experiments). **h** Time-lapse images of HeLa cells co-transfected with GFP-H2B and either siNC- or CCDC74A/B-siRNA. NEBD, nuclear envelope breakdown; Ana, anaphase. Numbers, time (min) after NEBD. Arrows, misaligned chromosomes. Scale bar, 5 μm. **i** Time elapsed from NEBD to anaphase onset in the HeLa cells from **h** (3 independent experiments). **j** Percentages of mitotic HeLa cells with chromosomal misalignments from **h**. 5/62, 5 cells with misalignment chromosomes in 62 cells transfected with siNC. 29/71, 29 cells with misalignment chromosomes in 71 cells transfected with siCCDC74B. In **c**, **d**, **f**, and **i**, data are mean ± SEM (unpaired two-tailed Student’s *t* test, ***P < 0.001, **P < 0.01, *P < 0.05). In **g**, data are mean ± SEM (one-way ANOVA test, **P < 0.01, *P < 0.05; n.s., not significant)

To search new mitotic regulators, we picked up a number of functionally uncharacterized proteins with coiled-coil domains which were screened by centrosome mass spectrometry [20]. We observed the subcellular localization of the candidates in HeLa cells and focused on CCDC74A and CCDC74B (CCDC74A/B). Human CCDC74A/B are functionally uncharacterized proteins. Both of them were highly conserved from *Ciona Savignyi* to *Homo Sapiens* (Additional file 1: Figure S1a). Their amino acid sequences displayed 97.45% congruence (Additional file 1: Figure S1b), suggesting similarities of their structures and biological functions. When CCDC74A/B were overexpressed in HeLa cells, they showed prominent spindle localization (Additional file 1: Figure S1c and d). Immunostaining endogenous CCDC74A/B in COS-7 and HeLa cells confirmed that CCDC74A/B were localized at spindles and central spindles during mitosis (Fig. 1a, Additional file 1: Figure S1e).

To reveal the function of CCDC74A/B in mitosis, we used siRNA to knockdown CCDC74A/B in HeLa cells (Fig. 1b, Additional file 1: Figure S1e and f). The mitotic index increased to 8.6% in the CCDC74A/B knockdown cells, compared to 3.8% in the control cells (Fig. 1c). Upon the release from a 90-min nocodazole arrest, the percentages of prometaphase cells in CCDC74A/B-depleted cells increased to 77%, compared to 52% of control cells (Fig. 1d). We then constructed two CCDC74A/B knockout HEK293T cells by using CRISPR/Cas9 approach (Fig. 1e) [21, 22]. The viability of CCDC74A/B knockout cells was much lower than that of the wild-type cells as shown by MTT assays (Fig. 1f). Flow cytometry results further showed that CCDC74A/B knockout increased the percentages of cells in G2/M phase (knock out (KO); KO-1, 19.3%; KO-2, 18.2%) compared to wild-type (wild-type (WT), 13.3%) (Fig. 1g, Additional file 2: Figure S2a). Collectively, these results indicate that CCDC74A/B depletion prolongs prometaphase and perturbs cell proliferation.

Anaphase will not start until all chromosomes are stably aligned by K-fibers at the equatorial plate. While without this step, cells will be arrested in prometaphase [23]. We used time-lapse microscopy to monitor chromosomal alignment in HeLa cells expressing the GFP-human histone 2B (H2B). Control cells spent ~ 59 min from nuclear envelope breakdown (NEBD) to the onset of anaphase and showed efficient chromosome congression (Fig. 1h, i; Additional file 7: Video S1). In contrast, in CCDC74A/B-depleted cells, the time from NEBD to anaphase onset took ~ 100 min and the ratio of cells with chromosomal misalignment significantly increased (Fig. 1h–j; Additional file 7: Video S1), suggesting that CCDC74A/B are required for chromosomal alignment.

### CCDC74A/B are required for spindle formation and integrity

**Fig. 2:**
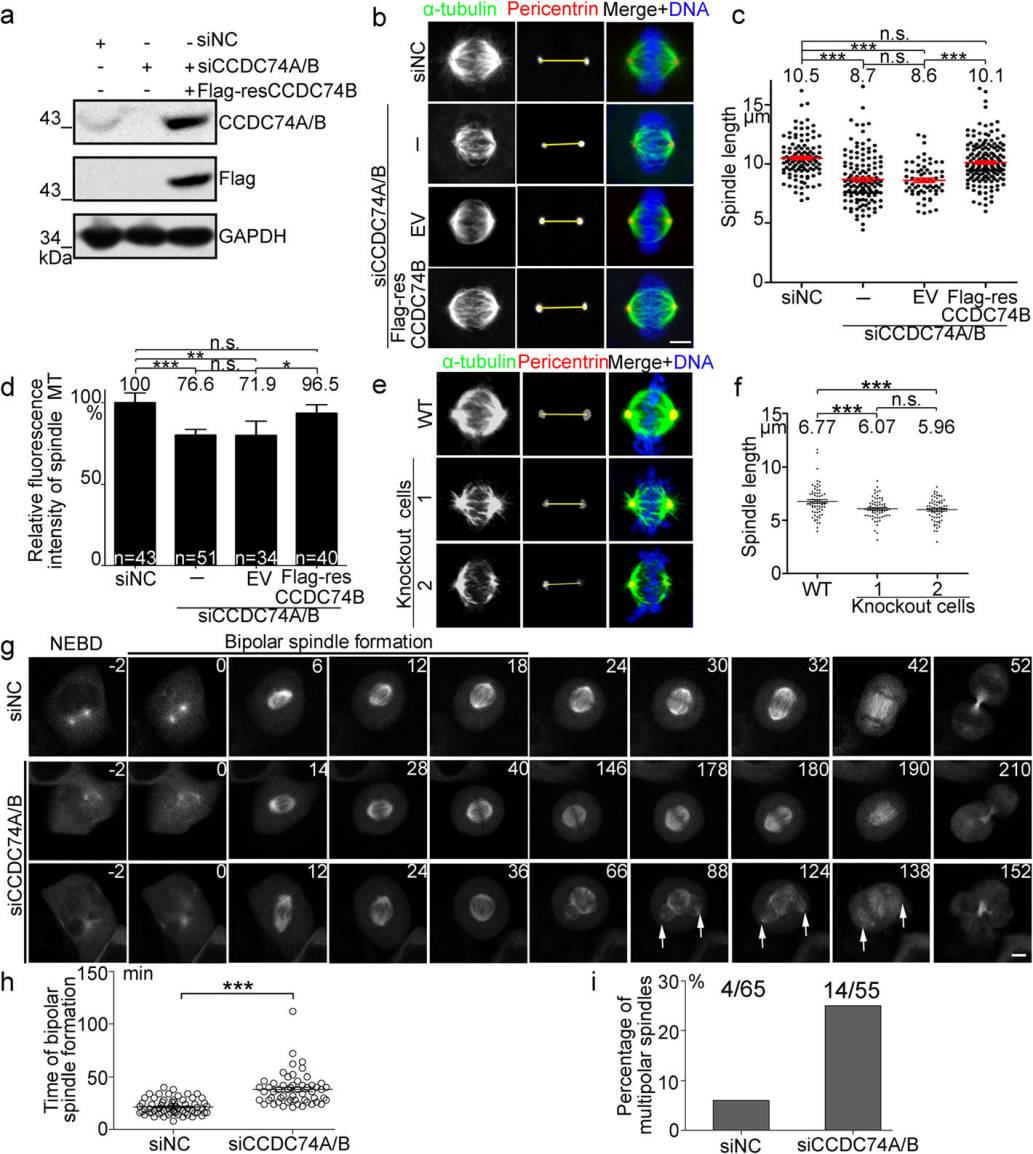
CCDC74A/B are required for spindle formation and integrity. **a** HeLa cells were transfected with negative control (siNC), CCDC74A/B-siRNA (siCCDC74A/B), or siCCDC74A/B together with siRNA-resistant Flag-CCDC74B (Flag-resCCDC74B) for 60 h. Western blots of endogenous CCDC74A/B and exogenous Flag-resCCDC74B (+, present; −, not present). GAPDH was loading control. **b**–**d** HeLa cells were transfected with siNC, siCCDC74A/B, or siCCDC74A/B together with empty vector (EV) or with Flag-resCCDC74B for 60 h. **b** Immunofluorescence of α-tubulin (green) and pericentrin (red). DNA was stained with DAPI (blue). Scale bar, 5 μm. **c** Statistical analysis of bipolar spindle length in cells from **b** (3 independent experiments). **d** Statistical analysis of fluorescence intensity of spindle microtubules (MT) in cells from **b**. The signal from siNC was normalized to 1.0 (3 independent experiments). **e** Immunofluorescence of α-tubulin (green) and pericentrin (red) in cells from the knockout HEK293T and wild-type (WT) cells. DNA was stained with DAPI (blue). Scale bar, 5 μm. **f** Statistical analysis of spindle lengths of cells from **e** (3 independent experiments). **g** Time-lapse images of HeLa cells co-transfected with GFP-tubulin and either siNC or siCCDC74A/B for 60 h. Numbers in each frame indicate the time (minutes) after nuclear envelope breakdown (NEBD). Arrows indicate abnormal spindle poles. Scale bar, 5 μm. **h** Time required for bipolar spindle formation in **g**. **i** Ratios of multipolar/normal mitotic cells in each group from **g**. 4/65, 4 cells with multipolar spindles in 65 cells transfected with negative control siRNA. 14/55, 14 cells with multipolar spindles in 55 cells transfected with CCDC74A/B siRNA. In **c**, **d**, and **f**, data are mean ± SEM (one-way ANOVA test, ***P < 0.001, **P < 0.01, *P < 0.05; n.s., not significant). In **h**, data are mean ± SEM (unpaired two-tailed Student’s *t* test, ***P < 0.001)

Since CCDC74A/B are localized at spindles (Fig. 1a and Additional file 1: Figure S1c-e) and their depletion impairs chromosomal alignment (Fig. 1h-j), we examined whether CCDC74A/B modulate mitotic spindle architecture. CCDC74A/B knockdown cells exhibited shorter spindles (~ 8.7 μm) compared to control cells (~ 10.5 μm), and spindle length was fully restored (~ 10.1 μm) after siRNA-resistant CCDC74B was introduced (Fig. 2a–c). Consistent with the reduction in spindle length, spindle microtubule fluorescence intensity also decreased by ~ 23% after CCDC74A/B knockdown (Fig. 2d). However, the introduction of siRNA-resistant CCDC74B stemmed the decrease by ~ 5% (Fig. 2b, d). Similarly, we also observed a decreased spindle length in CCDC74A/B knockout HEK293T cells (KO-1, 6.07 μm; KO-2, 5.96 μm; WT, 6.77 μm) (Fig. 2e, f). The percentages of both spindle misorientation (Additional file 2: Figure S2b and c) and multipolar spindles (Additional file 2: Figure S2d and e) were also slightly increased in CCDC74A/B-depleted cells, which were rescued by siRNA-resistant CCDC74B (Additional file 2: Figure S2b-e). Thus, CCDC74A/B are required for bipolar spindle structure formation.

To further explore the roles of CCDC74A/B in the spindle formation, we performed live-cell imaging by overexpressing GFP-tubulin in HeLa cells. With the depletion of CCDC74A/B, spindle formation was delayed, and significant defects in mitotic progression were observed (Fig. 2g–i; Additional file 8: Video S2). Taken together, these data indicate that CCDC74A/B are required for bipolar spindle formation and mitotic progression.

### CCDC74A/B promote spindle assembly and K-fiber stability

**Fig. 3:**
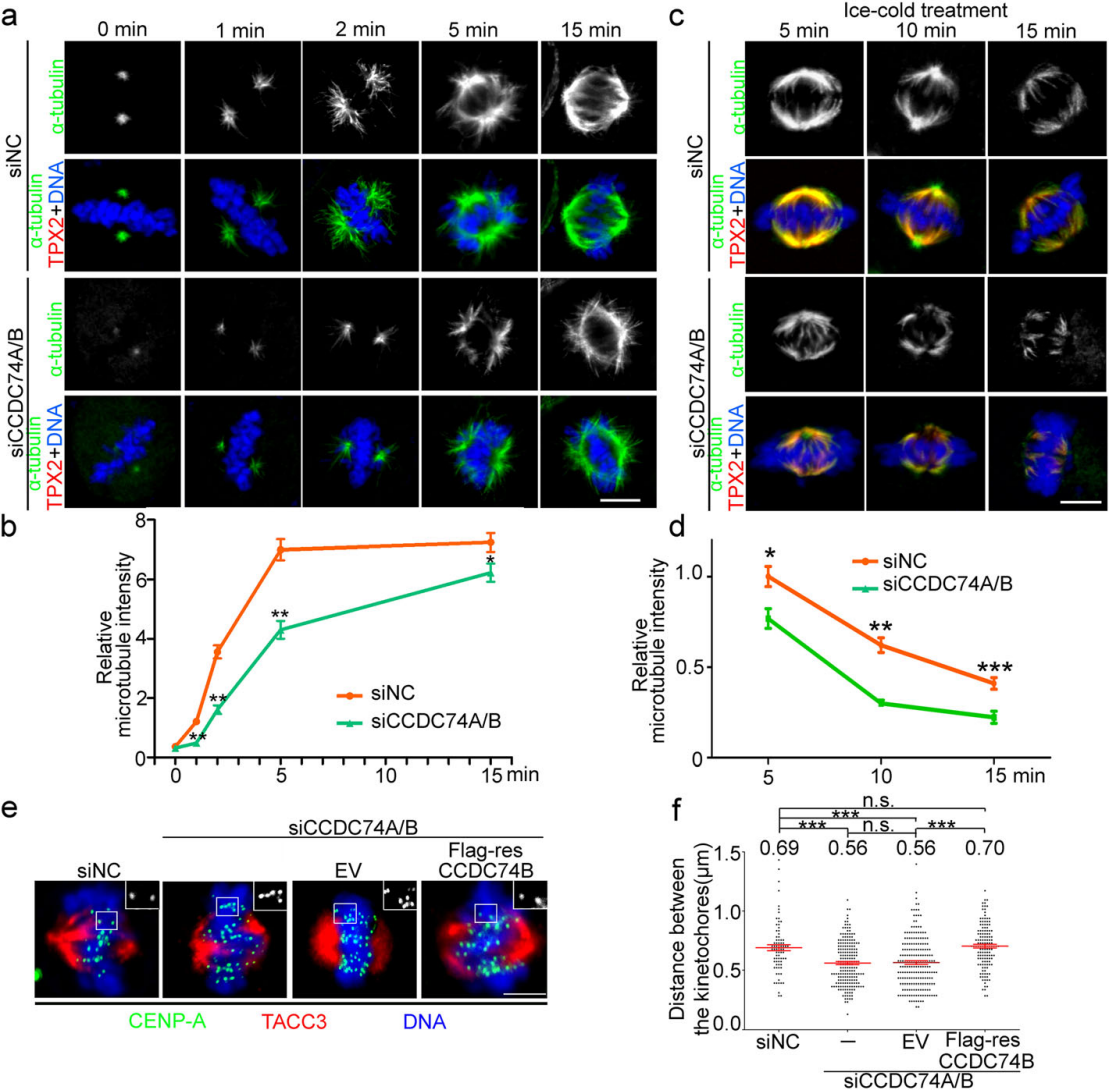
CCDC74A/B stabilize K-fibers. **a** Immunofluorescence of α-tubulin (green) in negative control (siNC) and CCDC74A/B-siRNA (siCCDC74A/B) HeLa cells after chilling 1 h on ice and rewarmed at 37 °C at the indicated time points. DNA was stained with DAPI (blue). Scale bar, 10 μm. **b** The microtubule signals from **a** were measured. Signals from siNC cells were normalized to 1.0 (three independent experiments). **c** Immunofluorescence of α-tubulin (green) and TPX2 (red) in siNC and siCCDC74A/B HeLa cells after ice chilling for the indicated time spans. DNA was stained with DAPI (blue). Scale bar, 10 μm. **d** Relative microtubule intensity from **c** (three independent experiments). **e** Immunofluorescence of CENP-A (green) and TACC3 (red) in siNC, siCCDC74A/B, or siCCDC74A/B together with empty vector (EV) or with siRNA-resistant Flag-CCDC74B (Flag-resCCDC74B) transfecting HeLa cells. DNA was stained with DAPI (blue). Scale bar, 5 μm. **f** Distances between kinetochores from **e** (three independent experiments). In **b** and **d**, data are mean ± SEM (unpaired two-tailed Student’s *t* test, ***P < 0.001, **P < 0.01, *P < 0.05). In **f**, data are mean ± SEM (one-way ANOVA test, ***P < 0.001; n.s., not significant)

To investigate whether CCDC74A/B regulate spindle assembly, we first examined the microtubule re-growth rate in interphase HeLa cells after a long, ice-cold treatment and allowed to recover by the addition of warm medium. Compared with the control cells, CCDC74B overexpression cells showed visible microtubule asters after 1 min of recovery, and cytoplasmic microtubules well recovered 5 min after regrowth (Additional file 3: Figure S3a and b). Then, we detected spindle microtubule re-assembly rate after recovering from cold treatment. Microtubule asters in the control cells grew and organized into bipolar spindles within 2 min and matured within 5 min; however, in the CCDC74A/B-depleted cells, the microtubule asters appeared delay and took 15 min to form bipolar spindles (Fig. 3a, b). These results suggest that CCDC74A/B promote bipolar spindle assembly.

To test whether CCDC74A/B affect microtubule stability, we chilled cells on ice to depolymerize spindle microtubules. After the 15-min cold treatment, only a few K-fibers remained and the shapes of bipolar spindles almost disappeared in CCDC74A/B-depleted cells (Fig. 3c, d). In contrast, a number of K-fibers were preserved, and bipolar spindle structures were still observed in control siRNA-treated cells (Fig. 3c, d). Consistently, depletion of CCDC74A/B resulted in fewer K-fibers after nocodazole treatment (Additional file 3: Figure S3c and d), whereas CCDC74B-overexpressed cells showed better spindle structure and K-fibers than wild-type cells at same time points after the cold treatment (Additional file 3: Figure S3e and f). Taken together, CCDC74A/B promote K-fiber microtubules stability.

Next, we examined whether CCDC74A/B knockdown changes the kinetochore tension generated by K-fibers [24, 25]. We measured the distance between opposing kinetochores (inter-kinetochore distance) in HeLa cells during metaphase. Inter-kinetochore distance decreased from ~ 0.69 μm in control cells to ~ 0.56 μm in CCDC74A/B knockdown cells, and the distance was restored (~ 0.7 μm) after siRNA-resistant CCDC74 is introduced (Fig. 3e, f), suggesting that CCDC74A/B deletion impairs the kinetochore microtubule attachment.

### CCDC74A/B possess microtubule-binding activity

**Fig. 4:**
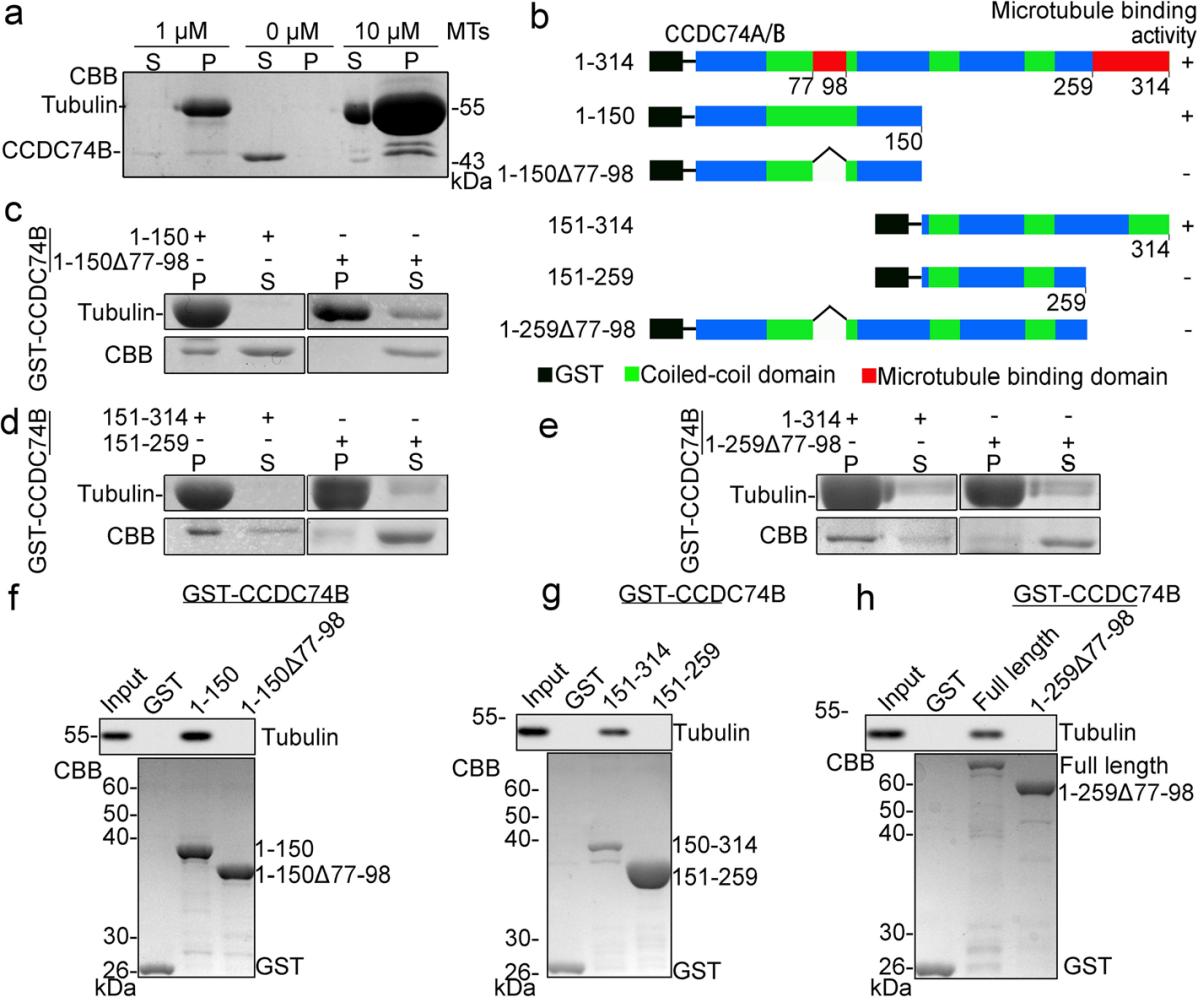
CCDC74A/B are microtubule-binding proteins. **a** Microtubule (MT) co-sedimentation assays in vitro. CCDC74B (0.2 μM) was expressed in *E. coli* then purified and incubated with or without taxol-stabilized microtubules in BRB80 buffer. After centrifugation, supernatants (S) and pellets (P) were separated and stained with Coomassie blue (CBB). **b** Schematic of GST-tagged CCDC74A/B full-length and their mutants, illustrating microtubule-binding activity of CCDC74B (+, positive; −, negative). **c**–**e** Western blot analysis of microtubule co-sedimentation assays in vitro. GST or GST-tagged full-length (1-314 aa) CCDC74B or the mutants in **b** were expressed in *E. coli*, purified, and incubated with taxol-stabilized microtubules in BRB80 buffer (+, present; −, not present). Supernatants and pellets were separated by centrifugation then stained with CBB. **f**–**h** GST or GST-tagged full-length CCDC74B or the indicated mutants (0.1 μM) bound to glutathione-sepharose 4B beads were incubated with taxol-stabilized microtubules in BRB80 buffer at room temperature in vitro. The bead-bound proteins were analyzed by Western blotting with anti-tubulin antibody and by CBB staining. GST served as a negative control

Since CCDC74A/B are well co-localized with spindle microtubules (Fig. 1a), we next examined whether they possess microtubule-binding activity by focusing on CCDC74B. First, microtubule co-sedimentation assays showed that *Escherichia coli* expressed and purified CCDC74B co-existed with microtubules in pellets in vitro (Fig. 4a). Then, to determine which regions are responsible for the microtubule co-sedimentation, we constructed a series of truncation and deletion CCDC74B mutants (Additional file 4: Figure S4a). Immunofluorescence assays revealed that two CCDC74B regions (79-98 aa and 260-314 aa) were independently responsible for spindle targeting (Additional file 4: Figure S4a and b). Next, to test whether the two regions contribute to the microtubule-binding, *E. coli* expressed GST-tagged full-length CCDC74B, and truncation or deletion mutants were purified and used in in vitro microtubule co-precipitation assays (Fig. 4b). The full-length, N- (1-150 aa) and C-termini (151-314 aa) of CCDC74B precipitated with microtubules in pellets, whereas the mutants lacking spindle-targeting regions (77-98 aa or 260-314 aa) appeared in the supernatants (Fig. 4b–e). We further performed pull-down assays by incubating *E. coli* expressed and purified GST-tagged full-length or mutant CCDC74B with assembled and taxol-stabilized microtubules in vitro. The full-length and N- and C-termini of CCDC74B, but not the mutants lacking microtubule-binding domains, were able to pull down microtubules (Fig. 4f–h). These results indicate that CCDC74A/B possess two microtubule-binding domains and each of them is sufficient to mediate microtubule binding.

### CCDC74A/B bundle microtubules in vivo and in vitro

**Fig. 5:**
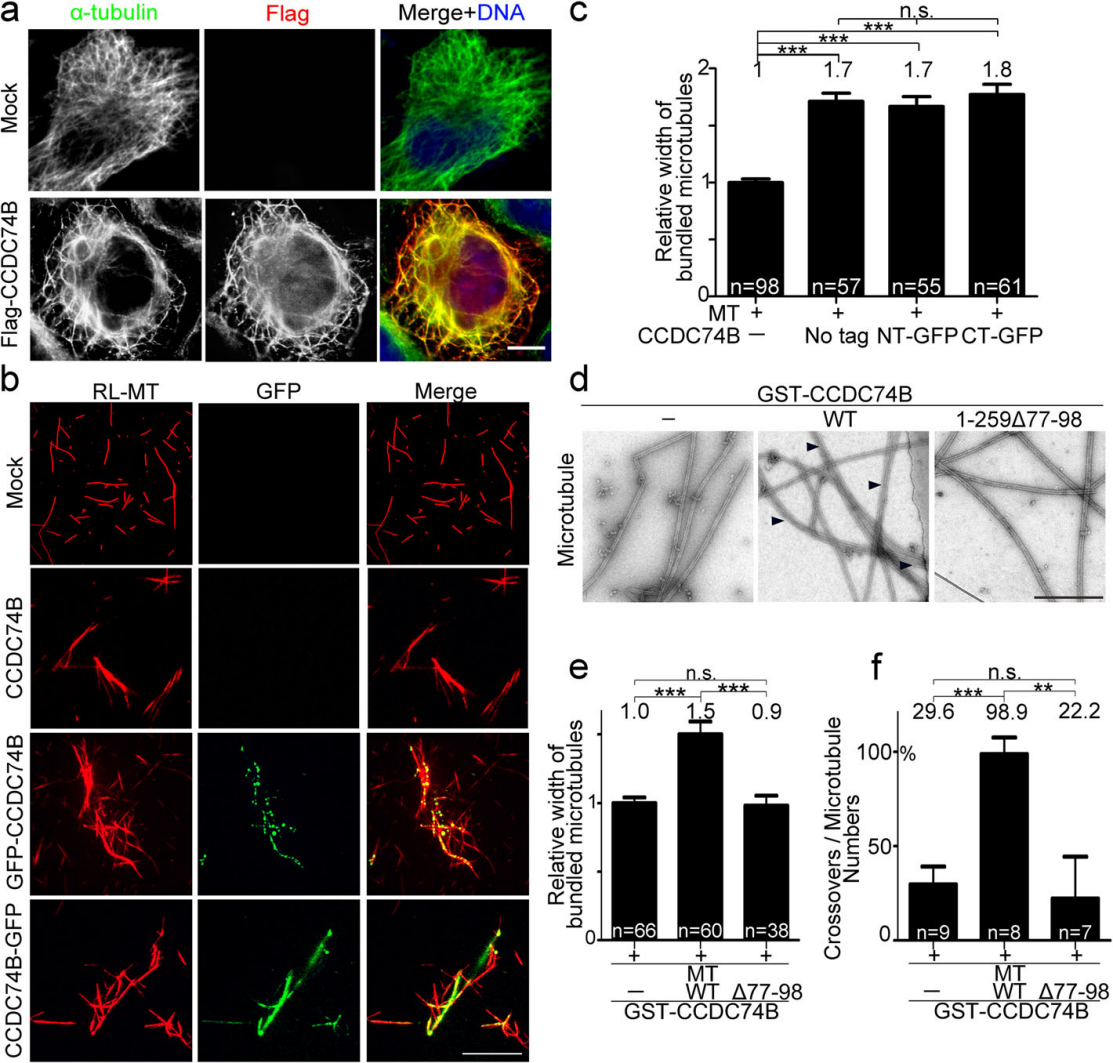
CCDC74A/B bundle microtubules in vivo and in vitro*.* **a** Immunofluorescence of Flag (red) and α-tubulin (green) in HeLa cells transfecting with or without Flag-CCDC74B for 24 h. DNA was stained blue with DAPI. Scale bar, 5 μm. **b** Wild-field images of purified CCDC74B, GFP-CCDC74B (green) or CCDC74B-GFP (green) incubated with rhodamine-labeled, and taxol-stabilized microtubules (RL-MT, red) (1 μM). Scale bar, 2 μm. **c** Statistical analysis of width of bundled microtubules in **b**. The mock group was normalized to 1.0. NT-GFP, N-terminal-tagged GFP, GFP-CCDC74B. CT-GFP, C-terminal-tagged GFP, CCDC74B-GFP (three independent experiments). **d** Negative-stained images of taxol-stabilized microtubules only (left), together with GST-CCDC74B wild-type (WT) (middle), or truncation mutant 1-259Δ77-98 (right) examined by electron microscopy. Scale bar, 500 nm. Black arrowheads indicate the bundled microtubule. **e** Statistical analysis of width of bundled microtubules in **d**. The mock group was normalized to 1.0 (three independent experiments). **f** Statistical analysis of crossovers/microtubules ratios in **d** (three independent experiments). In **c**, **e**, and **f**, data are mean ± SEM (one-way ANOVA test, ***P < 0.001, **P < 0.01; n.s., not significant)

We then determined how CCDC74A/B, as microtubule-binding proteins, regulate microtubule stability. Overexpression of CCDC74B induced microtubule bundling in the cytoplasm of HeLa cells (Fig. 5a). Similarly, in retinal-pigmented epithelial (RPE1) cells, overexpressed CCDC74A/B prompted microtubule bundling (Additional file 4: Figure S4c). The purified CCDC74B (with or without GFP-tag) was incubated with taxol-stabilized rhodamine-labeled microtubules in vitro, and microtubules were significantly bundled by CCDC74B (Fig. 5b, c; Additional file 4: Figure S4d). We further observed that the microtubules were bundled after the addition of wild-type GST-CCDC74B, but not of the mutant lacking the two microtubule-binding domains by electronic microscopy (Fig. 5d–f). These results illustrate that CCDC74A/B bundle microtubules and the two microtubule-binding domains are responsible for the bundling.

### CCDC74A/B own self-association activity

**Fig. 6:**
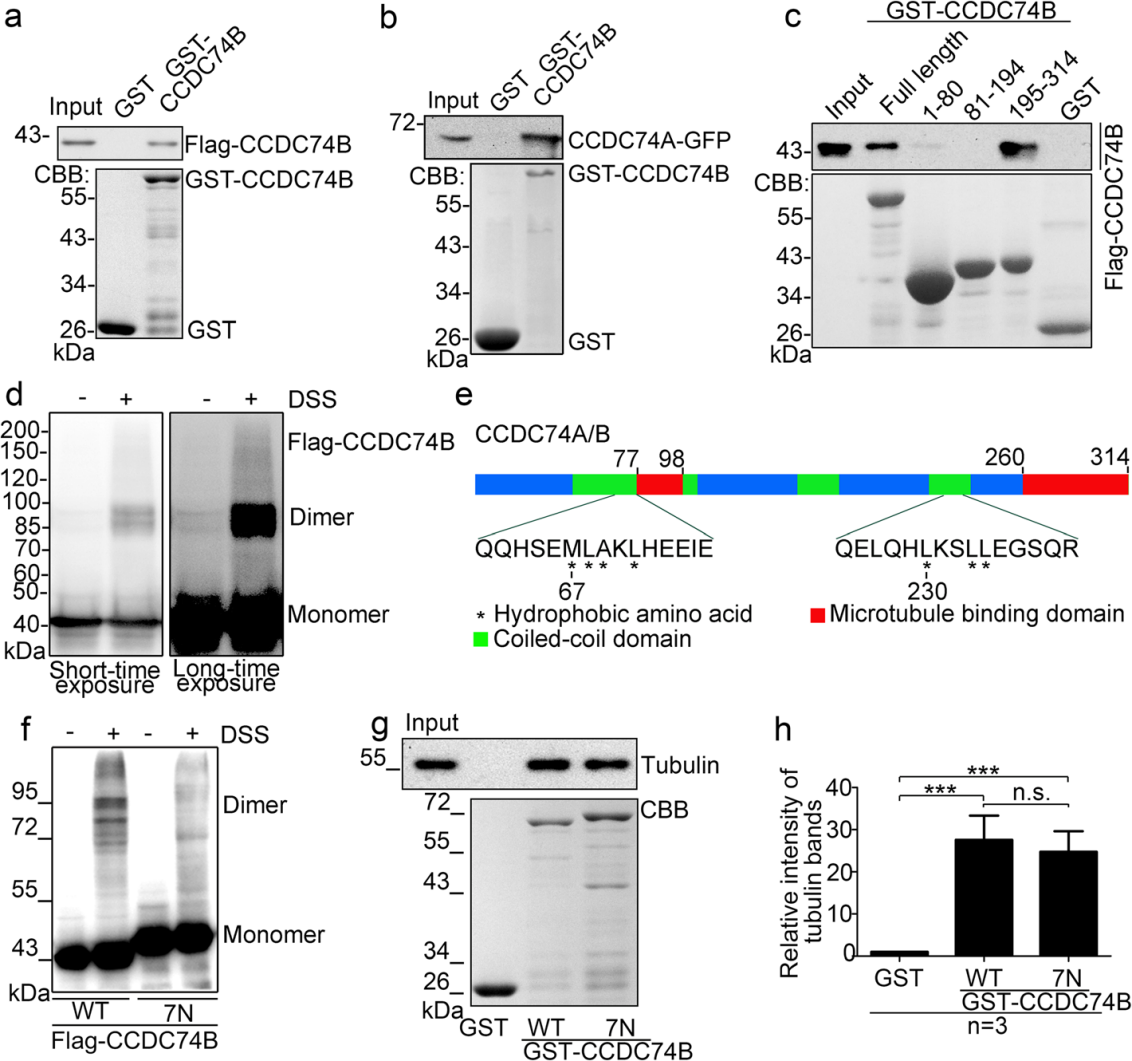
CCDC74A/B possess self-association activity. **a**–**c** GST pull-down assays. Flag-CCDC74B (**a**, **c**) or CCDC74A-GFP (**b**) (expressed in HEK293T cells) and GST-CCDC74B full-length or mutants (expressed in *E. coli* and purified) were incubated and then analyzed by Western blotting with anti-Flag (**a**, **c**) or anti-GFP (**b**) antibody and by Coomassie blue (CBB) staining. **d** Flag-CCDC74B-expressed HeLa cells were treated with 1 mM disuccinimidyl suberate (DSS) in DMSO for 30 min, then the samples underwent Western blotting with anti-Flag antibody. The long time exposure showed a dense band of CCDC74B dimers. **e** Schematic of human CCDC74B domains, illustrating seven amino acids required for CCDC74B dimerization. **f** Flag-CCDC74B wild-type (WT)- or 7N mutant-expressed HeLa cells were treated with 1 mM DSS in DMSO for 30 min. Samples were analyzed by Western blotting with anti-Flag antibody. 7N: MLAL67-71NNNN and LLL230-234 NNN. **g** GST-CCDC74B WT- or 7N mutant-coupled glutathione sepharose 4B beads were incubated in vitro with assembled, taxol-stabilized microtubules in BRB80 buffer at room temperature. The bead-bound proteins were analyzed by Western blotting with anti-tubulin antibody and by CBB staining. GST proteins served as a negative control. **h** Relative intensity of tubulin bands in **g**. The signal from GST group was normalized to 1.0. Data are mean ± SEM (one-way ANOVA test, ***P < 0.001; n.s., not significant; three independent experiments)

To determine whether CCDC74A/B have a self-association activity, we first purified Flag-CCDC74B from HEK293T cells and GST-CCDC74B from *E. coli* to perform the binding assays in vitro*.* GST-CCDC74B bound to Flag-CCDC74B (Fig. 6a). Likewise, purified CCDC74A-GFP from HEK293T cells bound to GST-CCDC74B from *E. coli* (Fig. 6b). Furthermore, we examined which regions of CCDC74B were responsible for its self-association. Pull-down assays using truncated mutants of GST-CCDC74B showed that the C-terminal region (195-314 aa) bound to Flag-CCDC74B, and the N-terminus (1-80 aa) also showed a very weak interaction (Fig. 6c). We further overexpressed Flag-CCDC74B in HeLa cells and then treated cells with the crosslinker disuccinimidyl suberate (DSS). Besides the monomers, we observed Flag-CCDC74B dimers based on the band size (Fig. 6d), indicating that overexpressed CCDC74B has the ability to form a dimer in vivo.

Next, we overexpressed several CCDC74B-truncated mutants in HeLa cells to further determine which region is responsible for the CCDC74B self-association. Only the middle region (99-220 aa) of CCDC74B did not form dimers (Additional file 5: Figure S5a). We analyzed the amino acid sequences of CCDC74A/B in the N- (1-98 aa) and C-terminals (221-314 aa) to identify the specific amino acids that mediated the association and found seven adjacent amino acid-containing regions (29-34 VVL, 51-53 LL, 57-60 LF, 67-71 MLAL, 75-78 IL, 230-234 LLL, and 282-286 LLL) (Fig. 6e). Next, we mutated the amino acids in these regions to asparagine (N). Both mutants MLAL67-71NNNN and LLL230-234NNN produced markedly reduced CCDC74B homodimerization (Additional file 5: Figure S5b). Furthermore, the mutant containing 7N (MLAL67-71NNNN and LLL230-234NNN) in full-length CCDC74B significantly lost its self-association ability (Fig. 6e, f). Size exclusion chromatography assay confirmed that the seven amino acid mutation did not change the protein folding of the CCDC74B as both wild-type CCDC74B and 7N mutant had the sharp main peak (Additional file 5: Figure S5c). We noticed that wild-type CCDC74B had a small peak eluted in the void volume, indicating that wild-type CCDC74B tended to form macromolecular aggregates in vitro and the 7N mutant decreased this self-association activity. Furthermore, microtubule co-sedimentation assay showed that 7N mutation did not significantly influence the microtubule-binding activity of CCDC74B (Fig. 6g, h; Additional file 9: Table S1).

Taken together, CCDC74A/B possess self-association activity, and their self-association ability is independent of their microtubule-binding activity.

### CCDC74A/B self-association is required for stabilizing microtubules and K-fibers

**Fig. 7:**
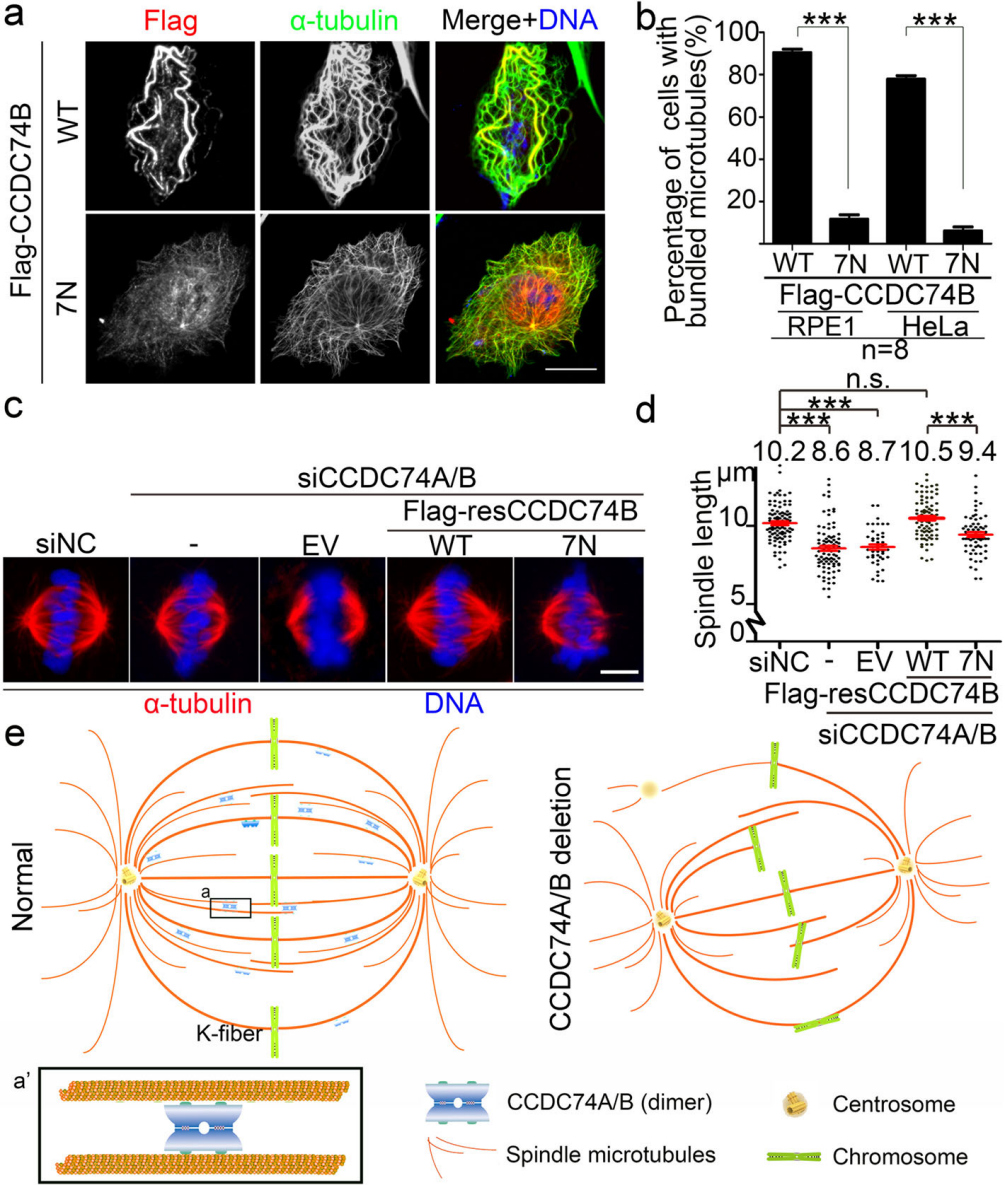
Self-association of CCDC74A/B is required for stabilizing microtubules and K-fibers. **a** Immunofluorescence of Flag (red) and α-tubulin (green) in RPE1 cells transfecting with Flag-CCDC74B wild-type or 7N mutant for 48 h (7N: MLAL67-71NNNN and LLL230-234 NNN). Scale bar, 20 μm. **b** Percentages of cells with bundled microtubules in **a** and in identically treated HeLa cells (three independent experiments). **c** Overexpressed empty vector (EV), Flag-CCDC74B wild-type (WT), or 7N mutant into HeLa cells introduced with negative control siRNA (siNC) or CCDC74A/B siRNAs (siCCDC74A/B) for 48 h. The cells were immunostained with α-tubulin (red) and the DNA stained with DAPI. Scale bar, 5 μm. **d** Statistical analysis of bipolar spindle length in cells from **c** (three independent experiments). **e** Graphic of CCDC74A/B functions in bundling microtubules and stabilizing spindle microtubules especially K-fibers. CCDC74A/B bind to and bundle microtubules through both their dual microtubule-binding regions and self-association. CCDC74A/B depletion causes spindle and K-fiber organization defects, thus inducing chromosomal misalignment and cell cycle delay. In **b**, data are mean ± SEM (unpaired two-tailed Student’s *t* test, ***P < 0.001). In **d**, data are mean ± SEM (one-way ANOVA test, ***P < 0.001; n.s., not significant)

Next, we determined whether the microtubule bundling activity of CCDC74A/B depends on their self-association activity. Both overexpressed wild-type and 7N mutant CCDC74B were co-localized with microtubules in RPE1 and HeLa cells (Fig. 7a, Additional file 6: Figure S6a), suggesting 7N mutant still binds to microtubules. However, the percentage of cells with bundled microtubules was significantly reduced in 7N mutant-overexpressed cells compared with that in wild-type cells (Fig. 7b), indicating that CCDC74B self-association activity is required for it to bundle microtubules. To test whether the association activity is also necessary for CCDC74B-stabilizing microtubules, we overexpressed wild-type or 7N mutant CCDC74B in RPE1 cells and then chilled the cells on ice to depolymerize the microtubules. After the 20-min cold treatment, we found more microtubule bundles remaining in wild-type CCDC74B interphase cells than in 7N mutant interphase cells (Additional file 6: Figure S6b), suggesting that self-association of CCDC74B is important to stabilize microtubules. Moreover, during metaphase, Flag-CCDC74B wild-type and 7N mutant both displayed spindle localization in HeLa cells (Additional file 6: Figure S6c). Wild-type CCDC74B rescued the shorter spindle (control, ~ 10.2 μm; Flag-CCDC74B WT, ~ 10.5 μm) caused by knockdown CCDC74A/B (CCDC74A/B knockdown, ~ 8.6 μm), but not the 7N mutant (Flag-CCDC74B 7N, ~ 9.4 μm) (Fig. 7c, d). Similar results were also detected in CCDC74A/B knockout cells (Additional file 6: Figure S6d and e). These results indicate that self-association of CCDC74A/B is required for K-fiber formation and spindle assembly.

## Discussion

In this paper, we show that CCDC74A/B physically bind to microtubules with two separate regions and bundle microtubules through their self-association activity. CCDC74A/B depletion causes spindle microtubule organization particularly K-fiber defects, which leads to the chromosomal misalignment and multipolar spindle formation (Fig. 7e). Therefore, CCDC74A/B, as new spindle and K-fiber regulators, are required for K-fiber stability, ensuring accurate chromosome alignment (Fig. 7e).

Human CCDC74A/B, encoded by two adjacent open reading frames in chromosome 2, are highly conserved proteins (Additional file 1: Figure S1a and b). They show nearly identical amino acid sequences (97.45% match) (Additional file 1: Figure S1b), and the antibody we used in this paper label both of them. It is nearly impossible for us to find a siRNA sequence that specifically targets one and not the other protein-encoding mRNA for degradation. Our results show that both CCDC74A/B are localized at spindles. They may function redundantly in the spindle assembly and K-fiber stability. Therefore, we propose that CCDC74A and CCDC74B are two identical copies of the same gene, duplicated during evolution.

CCDC74A/B contain 2 separate regions, 79-98 aa and 260-314 aa, that enable direct binding to microtubules, and there are 23 positively charged amino acid residues (1H, 5K, 2R in 77-98 aa; 1H, 8K, 6R in 260-314 aa) in these regions. Tubulin dimers have a glutamate-rich, highly negatively charged C-terminal tail (E-hook), which mediates binding to MAPs, lying outside the microtubule lattice [16]. Therefore, the positively charged amino acid residues located in 79-98 aa (1H, 5K, and 2R) and 260-314 aa (1H, 8K, and 6R) are very likely responsible for the CCDC74A/B binding to tubulins via mutual attraction between charges. Of the 2 regions, each of them is enough to mediate microtubule binding and spindle targeting, since deletion of either 1-150 aa or 151-314 aa could not abolish the binding of CCDC74B to microtubules (Fig. 4f–h). Consistently, the targeting of CCDC74B 1-259 aa and 99-314 aa to spindles was abolished by 77-98 aa deletion and 260-314 aa deletion, respectively (Additional file 4: Figure S4a and b). The detailed mechanisms underlying the potential collaboration between these two microtubule-binding domains are still unclear and need further investigation.

The GST pull-down results indicate that CCDC74A/B possess self-association activity (Fig. 6a–c). It has been reported that the self-association activity helps proteins to form oligomers, and even macromolecules [26, 27]. Our results by size exclusion chromatography assay show that a small fraction of wild-type CCDC74B tends to form macromolecular aggregates in vitro and that the 7N mutant decreases this self-association activity (Additional file 5: Figure S5c-e). However, it is still unclear what kind of oligomer endogenous CCDC74A/B form, even though our data show that overexpressed CCDC74A/B tend to form a dimer (Fig. 6d–f). We speculate that the dimerized CCDC74A/B could provide four microtubule-binding sites to form stable microtubule bundles, like PRC1 [28]; however, the detail mechanisms of how cells regulate the state of the endogenous CCDC74A/B are still needed to be investigated.

Mitotic spindles are relatively immense, up to 60 μm in length, but a cell usually has less than an hour to assemble an intact spindle [29]. To accomplish this, substantial even redundant MAPs are needed, so that the robust formation and attachment of K-fibers can be done to ensure successful chromosomal alignment and separation. Based on our study, CCDC74A/B may promote spindle integrity particularly by bundling K-fibers (Fig. 7e).

An essential bipolar spindle formation step is focusing on the minus-end of sorted microtubules toward spindle poles. In mammalian cells, only a quarter of spindle microtubule minus-ends are thought to be embedded into the centrosome [11]. By crosslinking microtubules, dimeric microtubule-binding protein NuMA and dynein-dynactin complex appear to be essential for focusing spindle poles. NuMA is considered a critical crosslinker of microtubules [11]; other proteins, such as CCDC74A/B, could also contribute to organizing the huge spindle apparatus. Like NuMA, CCDC74A/B also show dimerization in vivo. However, CCDC74A/B are relatively small molecules with two independent microtubule-binding domains each, making them unique for crosslinking microtubules. Two separate microtubule-binding regions cooperate with self-association and contribute to CCDC74A/Bs’ microtubule bundling and stabilizing functions, as well as to their maintenance of bipolar spindle architecture. CCDC74A/B are co-localized with the entire spindles but show high abundance on the whole K-fibers, suggesting that they may mainly stabilize the spindle apparatus by crosslinking microtubules of K-fibers, thus differing from spindle pole organizer NuMA. Considering that CCDC74A/B depletion induces multipolar spindle formation (Additional file 2: Figure S2d and e), they very likely help with microtubule focusing by crosslinking spindle microtubules. CCDC74A/B depletion also causes spindle misorientation (Additional file 2: Figure S2b and c), but this phenotype may not be directly caused by CCDC74A/B depletion since CCDC74A/B did not show any appreciable enrichment at astral microtubules (Fig. 1a). It can be indirectly induced by less-organized spindle poles and shriveled K-fibers.

Every mammalian kinetochore can be attached by 20–30 microtubules simultaneously, but most spindle microtubules are not long enough to range from spindle poles to kinetochores [5]. This means that microtubule bundling and crosslinking are important for proper K-fiber formation. Given that CCDC74A/B are distributed ubiquitously among K-fiber microtubules, and that shorter spindle length was induced by CCDC74A/B depletion, CCDC74A/B very likely crosslink and bundle whole spindle/K-fibers. This would promote connections of microtubules between kinetochores and centrosomes/spindle poles, but not to regulate plus-end dynamics, thus differing from how HURP functions [8, 30]. Our findings present a worthy argument to continue investigating why CCDC74A/B need 2 independent microtubule-binding domains, the mechanisms of how CCDC74A/B crosslink microtubules, and whether CCDC74A/B collaborate with other crosslinkers, like NuMA and HURP.

## Conclusions

We identify that uncharacterized CCDC74A/B target to mitotic spindles and directly bind to and bundle microtubules, which promotes K-fiber stabilization and mitotic spindle integrity. Furthermore, they possess self-association activity to bundle and stabilize microtubules and K-fibers. Our findings provide insight into the new MAP-mediated K-fiber crosslinking and spindle assembly.

## Methods

### Plasmid construction

The full-length cDNAs of CCDC74A and CCDC74B were amplified from the HeLa cell cDNA library. Full-length and truncated cDNAs were inserted into pEGFP-C2 (Clontech), pEGFP-N3 (Clontech), pET28a (Novagen), pGEX-6P-1 (Amersham Biosciences), p3×Flag-CMV-7.1 (Sigma), and pHM3 (a gift from Dr. Can Xie).

### Antibodies

The anti-CCDC74A/B rabbit antibody (TA344341, OriGene) was used for Western blotting (WB, 1:1000) and immunofluorescence (IF, 1:100). The other antibodies used for WB or IF are as follows: anti-acetylated tubulin (T7451, Sigma, RRID:AB_609894) (IF, 1:100), anti-Flag (clone M2, F3165, Sigma, RRID:AB_259529) (WB, 1:1000; IF, 1:100), anti-α-tubulin (clone DM1A, T9026, Sigma, RRID:AB_477593) (WB, 1:5000; IF, 1:500), anti-GAPDH (clone GAPDH-71.1, G9295, Sigma, RRID:AB_1078992) (WB, 1:1000), anti-green fluorescent protein (GFP; clone RQ2, D153-3, MBL, RRID:AB_591817) (WB, 1:100), anti-GST (sc-33613, Santa Cruz Biotechnology, RRID:AB_647588) (WB, 1:1000), anti-TACC3 (sc-376883, Santa Cruz) (IF, 1:200), anti-TPX2 (11741-1-AP, Proteintech, RRID:AB_2208895) (WB, 1:200; IF, 1:50), anti-γ-tubulin (T3559 and T6557, Sigma, RRID:AB_477575 and RRID:AB_477584) (IF, 1:200), and anti-Pericentrin (ab4448, Abcam, RRID:AB_304461) (IF, 1:1000). We used Alexa Fluor 488/568-conjugated goat anti-mouse/rabbit IgG (Alexa Fluor series, Molecular Probes) (IF, 1:200) as secondary antibodies for immunofluorescence and HRP-conjugated goat anti-mouse/rabbit IgG (Jackson ImmunoResearch) (WB, 1:5000) as secondary antibodies for Western blotting.

### Cell culture

HeLa (American Type Culture Collection, ATCC, CCL-2), COS7 (ATCC, CRL-1651), RPE1 (ATCC, CRL-4000), and HEK293T (ATCC, CRL-3216) cells were cultured in Dulbecco’s modified Eagle’s medium (Gibco) containing 10% FBS (Gibco or CellMax) at 37 °C under 5% CO_2_. PEI or Lipo fectamine™ 2000 (Invitrogen) were used according to the manufacturer’s instructions for cell transfection.

### RNA interference

We used siRNAs synthesized by Invitrogen for transfection at 100–120 nM by Lipofectamine 2000 or 3000 (Invitrogen). The sense strand sequence of negative control siRNA was 5′-UUCUCCGAACGUGUCACGU-3′. The CCDC74A/B siRNA sequence was 5′-GCUCCUUCAACAAGCAAGA-3′. The siRNA-resistant cDNA was cloned by PCR. The siRNA-targeted region of CCDC74A/B was mutated into 5′-GGTCGTTTAATAAACAGG-3′ from 5′-GCTCCTTCAACAAGCAAG-3′.

### CCDC74A/B knockout cell construction

For generating CCDC74A/B knockout HEK293T cells, target oligos (5′-GGTGACGGGGCGCCAGGCTA-3′ and 5′-GAGCGGGAGAGTACCTGGCGA-3′ for CCDC74A/B) were synthesized and ligated into a gRNA vector and then transfected into HEK293T cells together with Cas9 and pcDNA3.1 (puromycin resistance). One week later, puromycin-resistant cells were selected using flow cytometry (Beckman Coulter, MoFlo XDP). Western blot analysis identified successful knockout cells in the proliferated clones.

### FACS analysis of propidium iodide-stained cells

Cells were fixed in 70% ethanol (pre-cooled to − 20 °C) and washed with PBS two times. Then, the cells were spun down at 1000×*g* and collected, and the cell pellets were re-suspended in PBS containing 0.25% Triton X-100 for 15 min (on ice). After a spin down for 1 min at 1000×*g*, the pellets were re-suspended in PBS containing 10 μg/ml RNase and 20 μg/ml propidium iodide and incubated on ice for 30 min. The samples were analyzed by FACS machines (Beckman Coulter, MoFlo XDP). Data were handled by Flowjo-v10 software.

### GST pull-down and Western blotting

Cell lysates used for GST pull-down assays were obtained as follows: cells were lysed in a lysis buffer (150 mM NaCl; 1 mM MgCl_2_; 50 mM Hepes, pH 7.4; 1 mM EGTA; and 0.5% Triton X-100) containing protease inhibitors. After centrifugation for 10 min (20,000×*g* at 4 °C), the supernatants were collected. GST and GST-tagged proteins were expressed in *E. coli* (BL21 strain) and incubated with glutathione sepharose 4B beads (Amersham Biosciences) for purification. Then, the beads were washed five times with lysis buffer and incubated with the cell lysates for 4 h at 4 °C. Then, the beads were pelletized and washed five times, and the samples were boiled with loading buffer (with SDS) for 5 min [31].

For Western blotting, SDS-PAGE was used to separate the proteins, and then the proteins were transferred to a polyvinylidene difluoride membrane (Millipore). The membrane was sequentially incubated with primary antibodies and secondary HRP-conjugated antibodies (Jackson ImunoResearch).

### Protein purification

For GST fusion proteins, CCDC74B wild-type and indicated mutants were constructed into the pGEX-6p-1 vector and expressed in *E. coil* BL21 cells (Stratagene, 200131). Logarithmic phase BL21 cells were induced by IPTG (1 mM) and cultured overnight at 16 °C. Cells were harvested and then lysed in buffer (0.5% Tween-20, 20 mM Tris-HCl, 150 mM NaCl, 1 mM DTT, 5 mM EGTA, pH 7.5). Fusion proteins were purified by glutathione sepharose 4B beads (GE Healthcare).

For proteins without GST tag, purified GST-tagged CCDC74B wild-type and 7N mutant proteins were digested by HRV 3C Protease in cleavage buffer (50 mM Tris-HCl, 0.15 M NaCl, pH 7.0) to remove the GST tag.

### Microtubule pull-down assays and co-sedimentation assays

Microtubules were assembled at 37 °C for 30 min in BRB80 buffer (100 mM PIPES; 1 mM MgSO_4_; 2 mM EGTA; 1 mM GTP, pH 6.8) and then stabilized with taxol (20 μM).

For microtubule pull-down assays, the taxol-stabilized microtubules were incubated with bead-coupled GST or GST-CCDC74B proteins (expressed in *E. coli* and purified) in BRB80 buffer at room temperature for 1 h, and then the beads were washed three times with BRB80 buffer before boiling.

For microtubule co-sedimentation assays, maltose-binding protein-tagged CCDC74B proteins were expressed in *E. coli* and purified. After the maltose-binding protein was excised by PreScission Protease (Z03092-100, GenScript), CCDC74B protein was incubated with assembled microtubules and the samples were centrifuged at 100,000×*g* for 10 min at 25 °C. The supernatants and pellets were boiled and separated using SDS-PAGE.

### In vitro microtubule-binding assays

Flag-CCDC74B fusion proteins were expressed in HEK293T cells and immunoprecipitated with Protein A-Sepharose beads (Amersham Biosciences) and monoclonal antibodies (anti-Flag, Sigma) then washed five times with lysis buffer containing a high salt concentration (500 mM NaCl). GST-CCDC74B proteins were expressed in *E. coli* (BL21 strain) and were coupled with glutathione sepharose 4B beads (Amersham Bioscience) according to the manufacturer’s instructions. Then, the beads were washed five times with lysis buffer. Flag-CCDC74B was eluted using 3× Flag peptides.

For in vitro binding assays, approximately 0.2 μg of GST and GST-CCDC74B were coupled to the beads and incubated with 0.2 μg of purified Flag-CCDC74B, then the beads were washed five times with lysis buffer. The samples were boiled for 5 min and analyzed with Western blotting.

### Electron microscope negative staining

Negatively stained sample preparations and image acquisitions were described elsewhere [32]. Briefly, the samples were placed on a copper grid with a thin layer of carbon over the holes and left for 1 min while the proteins and the carbon layer interacted. Then, the protein solution was removed, and the sample was stained using 2% (w/v) uranyl acetate solution. Images were captured by a transmission electron microscope (FEI, Tecnai G2 20 Twin).

### In vitro microtubule bundling assay

Microtubules were prepared by incubating a 32-μM porcine brain tubulin mix containing 10% rhodamine-labeled tubulin with 20 μM taxol, 1 mM GTP, 4 mM MgCl2, and 4% DMSO. The mixture was polymerized in a 37 °C water bath for more than 30 min in the dark. Then, 400 μl of warm BRB80 buffer (containing 20 μM taxol) was added to stop the reaction. The sample was subjected to Airfuge centrifugation to collect microtubule seeds in the taxol-BRB80 buffer (containing 20 μM taxol). For in vitro microtubule assembly assays, these taxol-stabilized microtubules were centrifuged onto coverslips, with the indicated purified proteins and immediately subjected to confocal microscopy (LSM 510, Zeiss Oberkochen, Germany).

### Immunofluorescence and time-lapse microscopy

For immunofluorescence, cells were fixed and permeabilized in methanol for 7–10 min at − 20 °C and incubated at room temperature for 1 h with primary antibodies in PBS containing 3% bovine serum. They were then incubated with secondary antibodies and 1 μg/ml DAPI (Wako Pure Chemical Industries). A confocal microscope (LSM 710 NLO, Zeiss) equipped with a × 100/1.40 NA objective lens was used to observe fixed cells.

For time-lapse microscopic observations, a PerkinElmer UltraVIEW VoX spinning-disk confocal microscope (Nikon) equipped with a × 40/0.9 NA objective lens was used to snap the live cells. For time tracking, cells were put in a chamber at 37 °C under 5% CO_2_.

### Size exclusion chromatography assay

Purified CCDC74 wild-type and 7N proteins were eluted by size exclusion chromatography on a Superdex Increase 75 10/300 GL by GE Healthcare and equilibriated with PBS buffer (500 mM NaCl, 2.7 mM KCl, 10 mM Na_2_HPO_4_, 2 mM KH_2_PO_4_, and pH 7.4). The volume of the Superdex Increase 75 10/300 GL was 24 ml. Eluted fractions were analyzed by SDS-PAGE and detected by Coomassie blue staining. The catalog of the Gel Filtration Standard is 151-1901, BIO-RAD.

### Statistical analysis

Fluorescence intensity and bipolar spindle length were measured with ImageJ software (National Institutes of Health). For the spindle length measurements, only the cells with two pericentrin signals focused at the same focal plane were selected. Statistical analyses were performed with SPSS and GraphPad Prism 5 software. Unpaired two-tailed Student’s *t* test was used to determine if two sets of data are significantly different from each other. One-way ANOVA test was used to determine if three or more sets of data are significantly different from each other.

## Additional files

Additional file 1: **Figure S1.** CCDC74A/B are microtubule-associated proteins. (TIF 6802 kb)

Additional file 2: **Figure S2.** CCDC74A/B depletion results in cell cycle arrest, spindle mis-orientation and multi-polar spindles. (TIF 1956 kb)

Additional file 3: **Figure S3.** CCDC74A/B promote microtubule assembly and stabilize K-fiber. (TIF 19615 kb)

Additional file 4: **Figure S4.** CCDC74A/B bind to and bundle microtubules in vivo and in vitro*. (TIF 13228 kb)*

Additional file 5: **Figure S5.** CCDC74A/B own self-association activity. (TIF 3090 kb)

Additional file 6: **Figure S6.** Self-association activity of CCDC7A/B is required for stabilizing microtubules. (TIF 5780 kb)

Additional file 7: **Video S1.** CCDC74A/B are required for chromosomal alignment. (MP4 9638 kb) (MP4 8976 kb)

Additional file 8: **Video S2.** CCDC74A/B are required for mitotic spindle assembly. (MP4 8976 kb) (MP4 9638 kb)

Additional file 9: **Table S1.** Individual data values of Fig. 6h. (XLSX 8 kb)

## Data Availability

All data generated or analyzed during this study are included in this published article and its supplementary information files.

## Abbreviations

CCDC74A/B: Coiled-coil domain-containing protein 74A and 74B
MT: Microtubule
K-fiber: Kinetochore fiber
MAP: Microtubule-associated protein
WT: Wild-type
KO: Knockout
WB: Western blot
IF: Immunofluorescence
PVDF: Polyvinylidene fluoride
H2B: Histone H2B
NEBD: Nuclear envelope breakdown
DSS: Disuccinimidyl suberate
noc: Nocodazole
NC: Negative control
CBB: Coomassie blue staining

## References

[1] Karsenti E, Vernos I (2001). The mitotic spindle: a self-made machine. Science..

[2] Rieder CL (1981). The structure of the cold-stable kinetochore fiber in metaphase PtK1 cells. Chromosoma..

[3] Maiato H, Logarinho E (2014). Mitotic spindle multipolarity without centrosome amplification. Nat Cell Biol.

[4] Milunovic-Jevtic A, Mooney P, Sulerud T, Bisht J, Gatlin JC (2016). Centrosomal clustering contributes to chromosomal instability and cancer. Curr Opin Biotechnol.

[5] Clarke PR, Zhang C (2008). Spatial and temporal coordination of mitosis by Ran GTPase. Nat Rev Mol Cell Biol.

[6] Bayliss R, Sardon T, Vernos I, Conti E (2003). Structural basis of Aurora-A activation by TPX2 at the mitotic spindle. Mol Cell.

[7] Bird AW, Hyman AA (2008). Building a spindle of the correct length in human cells requires the interaction between TPX2 and Aurora A. J Cell Biol.

[8] Breuer M, Kolano A, Kwon M, Li CC, Tsai TF, Pellman D (2010). HURP permits MTOC sorting for robust meiotic spindle bipolarity, similar to extra centrosome clustering in cancer cells. J Cell Biol.

[9] Koffa MD, Casanova CM, Santarella R, Kocher T, Wilm M, Mattaj IW (2006). HURP is part of a Ran-dependent complex involved in spindle formation. Curr Biol.

[10] Sillje HH, Nagel S, Korner R, Nigg EA (2006). HURP is a Ran-importin beta-regulated protein that stabilizes kinetochore microtubules in the vicinity of chromosomes. Current Biol..

[11] Radulescu AE, Cleveland DW (2010). NuMA after 30 years: the matrix revisited. Trends Cell Biol.

[12] Harborth J, Wang J, Guethhallonet C, Weber K, Osborn M (1999). Self assembly of NuMA: multiarm oligomers as structural units of a nuclear lattice. EMBO J.

[13] Subramanian R, Ti SC, Tan L, Darst SA, Kapoor TM (2013). Marking and measuring single microtubules by PRC1 and kinesin-4. Cell..

[14] Subramanian R, Wilson-Kubalek EM, Arthur CP, Bick MJ, Campbell EA, Darst SA (2010). Insights into antiparallel microtubule crosslinking by PRC1, a conserved nonmotor microtubule binding protein. Cell..

[15] Polak B, Risteski P, Lesjak S, Tolic IM (2017). PRC1-labeled microtubule bundles and kinetochore pairs show one-to-one association in metaphase. EMBO Rep.

[16] Yokoyama H (2016). Chromatin-binding proteins moonlight as mitotic microtubule regulators. Trends Cell Biol.

[17] Lansky Z, Braun M, Ludecke A, Schlierf M, Wolde PRT, Janson ME (2015). Diffusible crosslinkers generate directed forces in microtubule networks. Cell..

[18] Yokoyama H, Rybina S, Santarella-Mellwig R, Mattaj IW, Karsenti E (2009). ISWI is a RanGTP-dependent MAP required for chromosome segregation. J Cell Biol.

[19] Raemaekers T, Ribbeck K, Beaudouin J, Annaert W, Van Camp M, Stockmans I (2003). NuSAP, a novel microtubule-associated protein involved in mitotic spindle organization. J Cell Biol.

[20] Andersen JS, Wilkinson CJ, Mayor T, Mortensen P, Nigg EA, Mann M (2003). Proteomic characterization of the human centrosome by protein correlation profiling. Nature..

[21] Ran FA, Hsu PD, Wright J, Agarwala V, Scott DA, Zhang F (2013). Genome engineering using the CRISPR-Cas9 system. Nat Protoc.

[22] Sternberg SH, Redding S, Jinek M, Greene EC, Doudna JA (2014). DNA interrogation by the CRISPR RNA-guided endonuclease Cas9. Nature..

[23] Bharadwaj R, Yu H (2004). The spindle checkpoint, aneuploidy, and cancer. Oncogene..

[24] Ravi M, Shibata F, Ramahi JS, Nagaki K, Chen C, Murata M (2011). Meiosis-specific loading of the centromere-specific histone CENH3 in Arabidopsis thaliana. PLoS Genet.

[25] Smith CA, Mcainsh AD, Burroughs NJ. Human kinetochores are swivel joints that mediate microtubule attachments. eLife. 2016;5:e16159. 10.7554/eLife.16159 PMC5050023 27591356

[26] Jiang H, Wang S, Huang Y, He X, Cui H, Zhu X (2015). Phase transition of spindle-associated protein regulate spindle apparatus assembly. Cell..

[27] Pereira C, Reis RM, Gama JB, Celestino R, Cheerambathur DK, Carvalho AX (2018). Self-assembly of the RZZ complex into filaments drives kinetochore expansion in the absence of microtubule attachment. Curr Biol.

[28] Mollinari C, Kleman JP, Jiang W, Schoehn G, Hunter T, Margolis RL (2002). PRC1 is a microtubule binding and bundling protein essential to maintain the mitotic spindle midzone. J Cell Biol.

[29] Wuhr M, Chen Y, Dumont S, Groen AC, Needleman DJ, Salic A (2008). Evidence for an upper limit to mitotic spindle length. Curr Biol.

[30] Wong J, Fang G (2006). HURP controls spindle dynamics to promote proper interkinetochore tension and efficient kinetochore capture. J Cell Biol.

[31] Zhou H, Wang T, Zheng T, Teng J, Chen J (2016). Cep57 is a Mis12-interacting kinetochore protein involved in kinetochore targeting of Mad1-Mad2. Nat Commun.

[32] Guan Zhe, Cai Tiantian, Liu Zhongmin, Dou Yunfeng, Hu Xuesong, Zhang Peng, Sun Xin, Li Hongwei, Kuang Yao, Zhai Qiran, Ruan Hao, Li Xuanxuan, Li Zeyang, Zhu Qihui, Mai Jingeng, Wang Qining, Lai Luhua, Ji Jianguo, Liu Haiguang, Xia Bin, Jiang Taijiao, Luo Shu-Jin, Wang Hong-Wei, Xie Can (2017). Origin of the Reflectin Gene and Hierarchical Assembly of Its Protein. Current Biology.

