# Supplementary figures and images for: CCDC74A/B are K-fiber crosslinkers required for chromosomal alignment
Source: BMC Biol. 2019 Sep 14;17:73. doi: 10.1186/s12915-019-0694-9 (PMC6744678; doi:10.1186/s12915-019-0694-9)

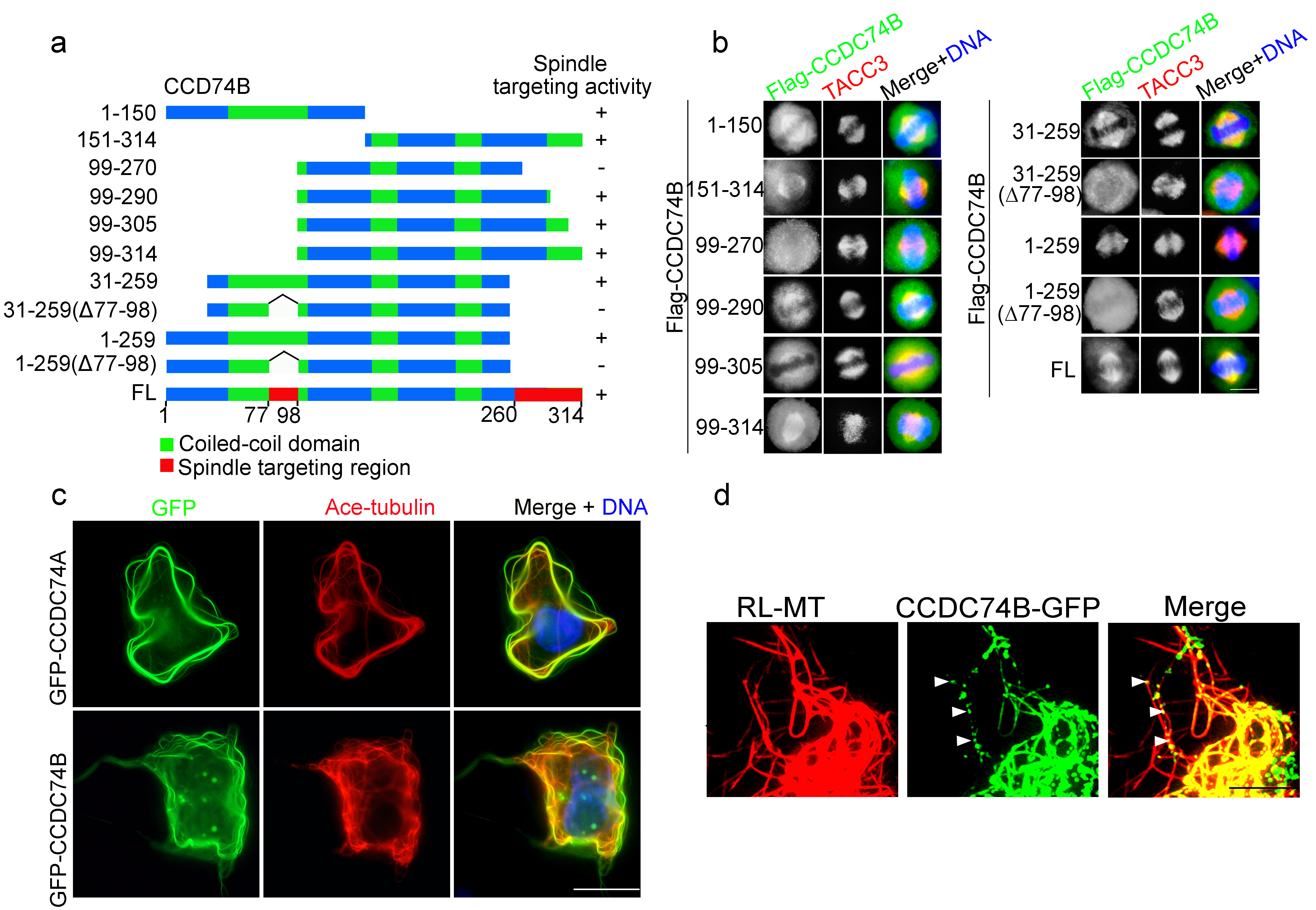

Supplement: Supplementary file 4 — Figure S4. CCDC74A/B bind to and bundle microtubules in vivo and in vitro. (TIF 13228 kb) [file 12915_2019_694_MOESM4_ESM.tif]
